# Efficacy and Safety of Nirmatrelvir/Ritonavir and Molnupiravir in Non-Hospitalized Patients with COVID-19: A Single-Center Retrospective Study

**DOI:** 10.3390/medicina62081566

**Published:** 2026-08-15

**Authors:** Maria Pete, Dajana Lendak, Nadica Kovačević, Aleksandra Pastornački, Milica Milovac, Vedrana Petrić

**Affiliations:** 1 Faculty of Medicine, University of Novi Sad, 21000 Novi Sad, Serbia; maria.pete@mf.uns.ac.rs (M.P.);; 2 Clinic for Infectious Diseases, University Clinical Center of Vojvodina, 21000 Novi Sad, Serbia; 3 Clinic for Anesthesia, Intensive Care and Pain Therapy, University Clinical Center of Vojvodina, 21000 Novi Sad, Serbia

**Keywords:** COVID-19, SARS-CoV-2, nirmatrelvir/ritonavir, molnupiravir, antiviral therapy, non-hospitalized patients

## Abstract

*Background and Objectives*: Oral antiviral agents nirmatrelvir/ritonavir (NMV/r) and molnupiravir are recommended for the early treatment of non-hospitalized adults with COVID-19 who are at increased risk of disease progression. Real-world comparative data on these two agents remain limited. The aim of this study was to evaluate the efficacy and safety of NMV/r and molnupiravir in non-hospitalized patients with COVID-19. *Materials and Methods*: We conducted a retrospective, single-center study with a sample size of 122 initially non-hospitalized adults with confirmed SARS-CoV-2 infection treated at the Clinic for Infectious Diseases, University Clinical Center of Vojvodina (UCCV), Novi Sad, Serbia, during 2022. Patients were treated within the first five days of symptom onset with either NMV/r (*n* = 48) or molnupiravir (*n* = 74) for five days. Demographic characteristics, clinical presentation, comorbidities, vaccination status, laboratory parameters at baseline and on day 3, chest radiography findings, hospitalization, and mortality were analyzed. Statistical analysis was performed using the Statistical Package for the Social Sciences (SPSS), version 21 (IBM Corp., Armonk, NY, USA). *Results*: The groups did not differ significantly in age, sex, vaccination status, or vaccine type. Between baseline and day 3, the increase in lymphocyte count (*p* < 0.001) was significantly greater in the molnupiravir group. Chest radiography showed no significant between-group differences. Overall, 6 patients (4.9%) were hospitalized, with no significant difference between antiviral agents (*p* = 1.000), and no deaths occurred. Among patient-reported symptoms effects, the incidence of dysgeusia was significantly higher in patients treated with NMV/r (*p* = 0.001). *Conclusions*: Both antivirals showed similarly low progression, hospitalization, and mortality rates.

## 1. Introduction

Severe acute respiratory syndrome coronavirus 2 (SARS-CoV-2) has posed a major global health problem, causing a prolonged pandemic characterized by recurrent waves of predominantly respiratory disease. The clinical manifestations of COVID-19 span a wide spectrum, ranging from asymptomatic infection to very severe disease with a fatal outcome [1]. Numerous studies have confirmed that individuals with comorbidities, including cardiovascular disease, chronic obstructive pulmonary disease (COPD), diabetes, chronic kidney disease, malignancy, and obesity, have a significantly increased risk of complications, prolonged disease, hospitalization, and mortality [1,2,3,4,5,6,7]. Since the beginning of the COVID-19 pandemic, the global scientific community has invested considerable effort in finding effective therapies against SARS-CoV-2, ranging from direct-acting antiviral drugs that inhibit viral replication within the host cell to immunomodulatory agents that regulate the excessive immune response [7]. The combination of different therapeutic measures has resulted in improved clinical outcomes in infected individuals. Vaccination, as well as early antiviral therapy initiated within the first five days of disease onset, has substantially reduced the risk of progression to severe disease and the risk of a lethal outcome [8]. Although SARS-CoV-2 infections are still present, both the disease and the virus have changed considerably. There is a strong possibility that, in the future, humanity will face an epidemic caused by coronaviruses again, as already happened with SARS in 2002 and MERS in 2012 [9]. Given that new COVID-19 variants continue to emerge, the role of antiviral therapy remains highly important and should not be neglected [10].

Nirmatrelvir/ritonavir and molnupiravir are oral antiviral drugs and represent the main therapeutic options for adults with COVID-19 who are at an increased risk of severe disease [11]. Molnupiravir is a prodrug that inhibits SARS-CoV-2 viral replication by introducing lethal mutations into the viral RNA, thereby preventing the further production of infectious particles [12]. It targets the enzyme RNA-dependent RNA polymerase (RdRp), which the coronavirus uses for transcription and replication of its viral RNA genome [13]. Nirmatrelvir is a potent, selective inhibitor of the SARS-CoV-2 main protease that is administered orally together with ritonavir (nirmatrelvir/ritonavir [NMV/r]; Paxlovid) in order to improve its pharmacokinetic profile. It has been shown to shorten the duration of COVID-19 symptoms and signs, as well as reduce the need for hospitalization and the use of healthcare services [14].

The aim of this study was to evaluate the efficacy and safety of nirmatrelvir/ritonavir and molnupiravir in non-hospitalized patients with COVID-19.

## 2. Materials and Methods

### 2.1. Study Design

This retrospective study included 122 initially non-hospitalized patients with a confirmed COVID-19 diagnosis by reverse transcription polymerase chain reaction or rapid antigen testing using a nasopharyngeal swab.

The study was conducted at the Clinic for Infectious Diseases, University Clinical Center of Vojvodina (UCCV), Novi Sad, Republic of Serbia, from January to December 2022. It was performed in accordance with the ethical principles of Good Clinical Practice and was approved by the Ethics Committee of the UCCV (approval number 00-412).

Data were collected retrospectively from electronic healthcare records for all consecutive patients diagnosed with COVID-19.

### 2.2. Patient Selection

Inclusion criteria were age over 18 years and confirmed SARS-CoV-2 infection. Exclusion criteria were symptom duration exceeding five days, pregnancy, hospitalization at the time of assessment, and refusal to receive antiviral therapy.

Therapeutic management was conducted in accordance with the European Medicines Agency (EMA) recommendations available during the study period. Decisions regarding the initiation of antiviral therapy were based on the contemporaneous EMA guidance and clinical eligibility criteria [15]. Decisions regarding the initiation of antiviral therapy were made according to physician evaluation and clinical assessment, patient comorbidities, potential drug–drug interactions, and eligibility criteria, following the contemporaneous EMA guidance. No patients were excluded due to missing data.

**Figure 1:**
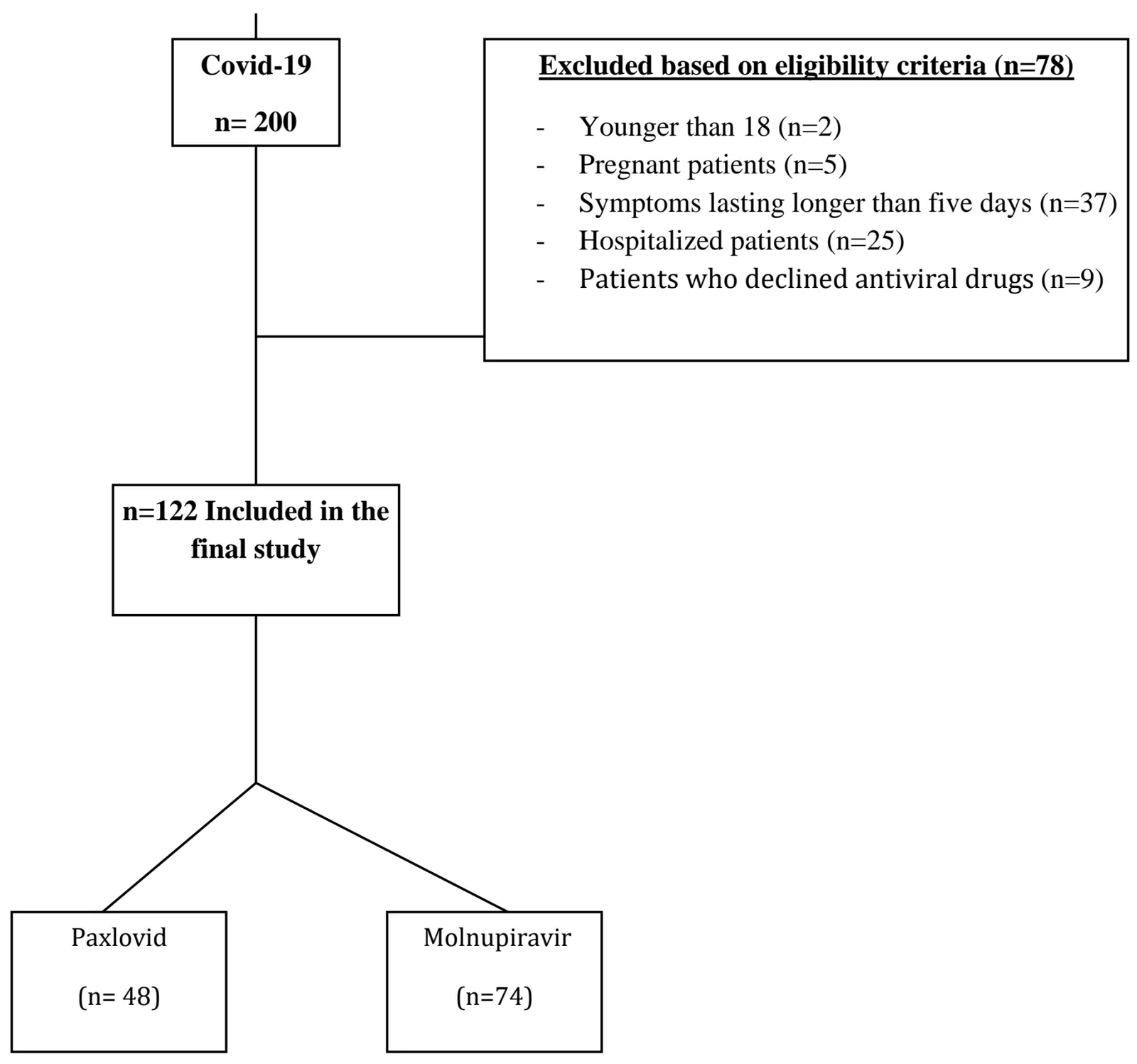
Patient selection flowchart.

A total of 122 patients were included in the final analysis and divided into two groups according to the antiviral drug received. The first group comprised 48 patients treated with paxlovid (39.3%), whereas the second group comprised 74 patients treated with molnupiravir (60.7%) (Figure 1). Both treatment options were initiated within the first five days of illness and administered for five days at the recommended doses. The dosages were as follows: 300 mg of nirmatrelvir (two tablets) and 100 mg of ritonavir (one tablet) administered every 12 h for five days and 800 mg of molnupiravir (four capsules) administered every 12 h for five days.

### 2.3. Laboratory Testing

For all patients included in the study, demographic data, clinical characteristics, COVID-19 vaccination history, comorbidities, chest radiography findings, hospitalization, and mortality were analyzed. Routine laboratory tests were performed for all the patients at the time of antiviral drug prescription and on day 3 of therapy. The analyzed laboratory parameters included complete blood count (CBC) with white blood cell count (WBC), neutrophil count (Neu), lymphocyte cell count (Lymph), sodium (Na), potassium (K), glucose, urea, creatinine, C-reactive protein (CRP), procalcitonin (PCT), alanine aminotransferase (ALT), aspartate aminotransferase (AST), and gamma-glutamyl transferase (GGT). All-cause mortality at day 28 was evaluated via telephone contact with patients or their caregivers.

### 2.4. Statistical Analysis

Statistical analysis was performed using the Statistical Package for the Social Sciences (SPSS), version 21 (IBM Corp., Armonk, NY, USA). Continuous variables with a normal distribution are presented as the mean (SD), whereas variables with a non-normal distribution are presented as the median (IQR). Categorical variables are presented as frequencies and percentages (n (%)). The normality of data distribution was assessed using the Shapiro–Wilk test. Comparisons between groups were performed using the independent-samples t-test for normally distributed continuous variables and the Mann–Whitney U test for variables that did not meet the assumption of normality. For variables with a large number of tied values, the exact Mann–Whitney U test was applied. Differences in the frequencies of categorical variables were assessed using the chi-square (χ^2^) test or Fisher’s exact test, as appropriate. To control for type I error arising from multiple comparisons, a Bonferroni correction was applied to the comorbidity analysis across eight comparisons, yielding a corrected significance threshold of α = 0.05/8 = 0.00625. For comparisons of laboratory parameters (16 tests in total), the Benjamini–Hochberg procedure was applied to control the false discovery rate (FDR) at q = 0.05. Procalcitonin (PCT) was excluded from this set of comparisons owing to a high proportion of tied values and was instead analyzed separately using the exact Mann–Whitney U test (two-sided exact *p* = 0.127). A two-sided *p*-value < 0.05 was considered statistically significant before correction for multiple comparisons. The results are presented in tables with accompanying textual interpretation.

## 3. Results

The study included 122 non-hospitalized patients with confirmed SARS-CoV-2 infection within the first five days of disease onset. Of these, 57 (46.7%) were male, and 65 (53.3%) were female. The mean age of the patients was 62.40 years (SD 13.147), with the youngest patient aged 23 years and the oldest aged 96 years. There were no statistically significant differences between the groups in terms of sex (χ^2^ test; χ^2^ = 0.812; *p* = 0.367) or age (Mann–Whitney U test; U = 142.7000; *p* = 0.067). For better differentiation between age categories, patients were divided into two age groups (18–65 years and >65 years old). Pearson’s chi-square (χ^2^) test demonstrated no statistically significant association between age category and the type of administered drug (χ^2^ = 0.702; df = 1; *p* = 0.402). The obtained *p*-value exceeded 0.05, indicating that the age distribution did not differ significantly between the groups treated with nirmatrelvir/ritonavir and molnupiravir. Overall, 100 patients (82%) had been vaccinated against SARS-CoV-2 infection. No statistically significant difference was observed in the prescribed drug with respect to patient vaccination status or the vaccine type. Baseline demographic characteristics are presented in Table 1.

The clinical presentation and comorbidity presence were further analyzed. The most frequently reported symptoms were weakness, malaise, elevated body temperature, and cough, whereas cardiovascular diseases were the most prevalent comorbidities. No statistically significant differences were observed in the distribution of comorbidities between the treatment groups. The results are presented in Table 2 and Table 3.

The mean values of the observed laboratory test parameters are summarized in Table 4.

Routine laboratory tests performed at the time of antiviral therapy initiation and on day 3 of treatment were analyzed. The Shapiro–Wilk test indicated a significant deviation from normality in the observed laboratory parameters; therefore, non-parametric tests were used for further comparison. These results are presented in Table 4. Chest radiographic findings were evaluated at the initial examination and on day 3 after the initiation of antiviral therapy. No statistically significant difference was found between the treatment groups in the frequency of pneumonia at the control examination compared with the initial examination. Likewise, no statistically significant difference was found in the degree of regression of previously confirmed pneumonia at the control examination between the antiviral agents used (Table 5).

Overall, 6 (4.9%) patients were hospitalized, including 2 (4.2%) treated with paxlovid and 4 (5.4%) treated with molnupiravir. There was no statistically significant difference in hospitalization rates according to the type of drug administered (*p* = 1.000). There were no deaths in the study sample. In our study, there were no statistically significant differences in outcome according to the type of antiviral drug administered, vaccination status, or vaccine type. These results are presented in Table 6.

For the comparison of hospitalization between treatment groups, the odds ratio (OR) was 0.76 (95% CI: 0.13–4.30) and the relative risk (RR) was 0.77 (95% CI: 0.15–4.05). The risk difference (RD) was −1.24% (95% CI: −8.9% to 6.4%), corresponding to an absolute risk reduction (ARR) of 1.24% and a relative risk reduction (RRR) of 22.9%. Fisher’s exact test was applied, yielding *p* > 0.05, indicating no statistically significant difference in hospitalization risk between the two treatment groups. For the comparison of hospitalization according to vaccination status, the odds ratio (OR) was 0.31 (95% CI: 0.02–5.63) and the relative risk (RR) was 0.34 (95% CI: 0.02–5.85). The risk difference (RD) was −6.0%, corresponding to an absolute risk reduction (ARR) of 6.0% and a relative risk reduction (RRR) of approximately 66%. Fisher’s exact test was applied, yielding *p* = 0.590, indicating no statistically significant difference in hospitalization risk between vaccinated and unvaccinated patients. The wide confidence intervals reflect the limited number of hospitalization events and should be interpreted with caution.

In the overall cohort, dysgeusia (9.8%) and diarrhea (9.0%) were the most frequently patient-reported symptoms. The incidence of dysgeusia was numerically higher in patients treated with NMV/r (20.8%) than in those receiving molnupiravir (2.7%); although this difference reached nominal statistical significance (*p* = 0.001), the small absolute number warrants cautious interpretation, and this finding should be considered hypothesis-generating rather than conclusive. No statistically significant intergroup differences were observed for the remaining symptoms, including diarrhea, headache, nausea, and vomiting (*p* > 0.05). Headache was numerically more frequent in the molnupiravir group (4.1% vs. 2.1%); however, given the small number of cases, this difference should be interpreted with caution and may reflect chance variation rather than a true treatment-related effect. Furthermore, a review of symptoms documented at the initial examination revealed that similar complaints had already been present at baseline in several patients, and it was therefore not possible to reliably distinguish treatment-emergent symptoms from those attributable to the underlying disease. These results are presented in Table 7.

## 4. Discussion

Our retrospective study included 122 initially non-hospitalized patients with confirmed SARS-CoV-2 infection who received antiviral therapy with either molnupiravir or nirmatrelvir/ritonavir. The initiation of antiviral therapy in the treatment of COVID-19 has had a significant impact on the course of the disease and patient management [16]. This study included patients with SARS-CoV-2 infection within the first five days of illness who had at least one risk factor for a more severe disease progression. Patients were divided into two groups according to the antiviral therapy: one received nirmatrelvir/ritonavir and the other received molnupiravir. The mean age of the patients was 62.40 years (SD 13.147), with the youngest patient aged 23 and the oldest aged 96 years. In our study, no statistically significant difference in age was observed between the two groups of patients, consistent with the findings of other studies [17,18]. In contrast, Gentile et al. reported that patients who received nirmatrelvir/ritonavir were younger [16]. Most participants had been vaccinated against SARS-CoV-2.

In our study, the most frequently reported symptoms were weakness, malaise, elevated body temperature, and cough. The most common comorbidities were cardiovascular diseases, affecting 53 (43.4%) patients, which is consistent with previously published data identifying cardiovascular disease, together with diabetes mellitus and chronic respiratory disease, as the most common comorbidities among individuals at increased risk of a more severe course of COVID-19 [2,19,20]. No statistically significant differences were observed in the distribution of comorbidities between the treatment groups. Patients with metabolic and cardiovascular comorbidities, as well as older patients, received molnupiravir more often, primarily because it has fewer clinically significant drug–drug interactions than nirmatrelvir/ritonavir [11,21,22]. This finding likely reflects a clinical prescribing pattern in which molnupiravir, owing to the absence of CYP3A4 inhibition, is considered a more suitable option for polymorbid patients receiving chronic therapy.

Analysis of the clinical presentation demonstrated statistically significant differences in the distribution of certain gastrointestinal symptoms between the two treatment groups. Vomiting (*p* = 0.015) and diarrhea (*p* = 0.033) were significantly more frequent among patients treated with nirmatrelvir/ritonavir, whereas weakness and malaise (*p* = 0.001) were more common in those receiving molnupiravir. In an effort to obtain a more robust basis for comparison, baseline symptomatology was recorded prior to the initiation of treatment. This methodological approach was adopted with the aim of facilitating a more nuanced interpretation of patient-reported symptoms observed after treatment initiation, potentially allowing for a tentative distinction between manifestations attributable to COVID-19 infection itself and those possibly related to therapy-associated effects [23,24,25].

Regarding laboratory parameters, comparison of changes in values from the initial measurement and the follow-up assessment on day 3 demonstrated statistically significant differences in lymphocyte count (*p* < 0.001), with a greater increase in lymphocyte count observed in the group treated with molnupiravir. Recovery of lymphocyte count has been recognized as one of the earliest and most sensitive indicators of clinical improvement in COVID-19, as lymphopenia is closely associated with disease severity, while normalization of the lymphocyte lineage precedes both radiological and virological recovery [26,27,28].

Chest radiographic analysis showed no statistically significant differences between the treatment groups, either in the frequency of pneumonia at treatment initiation or in the degree of regression of previously confirmed pneumonia at the follow-up examination. This is consistent with the results of studies comparing the two oral antiviral agents, which likewise reported no significant differences in disease progression or radiological outcomes with early administration in patients with mild-to-moderate disease [21]. The absence of radiographic progression in either group further supports the notion that early antiviral therapy, initiated within the first five days of symptom onset, is associated with a favorable disease course regardless of the drug choice.

Regarding outcomes, only 6 (4.9%) patients in our sample were hospitalized, and none died. Furthermore, no statistically significant difference in the hospitalization rates was observed between the two drugs (*p* = 1.000). The low hospitalization rates and the absence of deaths are consistent with findings from previous real-world observational studies, in which early administration of either oral antiviral agent to high-risk non-hospitalized patients was associated with low rates of disease progression, hospitalization, and mortality [11,21,29,30]. Although several large cohort and meta-analytic studies have suggested that nirmatrelvir/ritonavir may have a more pronounced effect in reducing the risk of hospitalization than molnupiravir, these differences are most pronounced in unvaccinated and older individuals, whereas the absolute benefit of either drug becomes considerably smaller in vaccinated individuals younger than 65 years [8]. Since 82% of our patients had been vaccinated against SARS-CoV-2, the absence of a difference in outcome between the groups may partly reflect the high vaccination coverage, which in itself lowers the baseline risk of a more severe disease course. Accordingly, in our study, we found no statistically significant association between outcomes and either vaccination status or vaccine type. The tolerability profile observed in our cohort was broadly favorable across both treatment options. Dysgeusia and diarrhea were the most frequently reported symptoms (9.8% and 9.0%, respectively), with dysgeusia occurring significantly more often among patients treated with NMV/r (20.8% vs. 2.7%; *p* = 0.001), consistent with previously reported data. Although headache was numerically more prevalent in the molnupiravir group (4.1% vs. 2.1%), this difference did not achieve significance, a result that may be attributable to the relatively small number of participants. No significant between-group differences were observed for the remaining symptoms, including diarrhea, headache, nausea, and vomiting (*p* > 0.05). Notably, all symptoms were mild and self-limiting [25]. The small number of patients who reported symptoms during antiviral treatment limits the statistical power of this analysis and substantially increases the risk of both false-positive and false-negative findings. These results should therefore be interpreted with considerable caution.

Our study has several limitations. First, this was a retrospective, single-center study with a relatively small sample size and without a control group that did not receive antiviral therapy, which precluded the assessment of the absolute efficacy of the drugs relative to no treatment. Furthermore, patient allocation to treatment groups was not randomized but based on clinical judgment and the presence of comorbidities, leading to imbalances between the groups (particularly in the prevalence of diabetes) and introducing a risk of treatment selection bias. In addition, genotyping of circulating viral variants was not performed. Because the data were collected during 2022, a period of Omicron strain dominance, the results cannot be uncritically extrapolated to future variants. The sampled cohort was skewed towards older age (average of >60 years old) and disproportionate numbers between NMV/r (N = 48) and molnupiravir (N = 74) treatment groups. Given these limitations, further validation of the findings reported here is warranted. The primary aim of this study was exploratory, and despite these limitations, our results provide insight into the efficacy and safety of the two most commonly used oral antiviral drugs under real-world clinical conditions and may serve as a foundation for further prospective, multicenter studies.

## 5. Conclusions

In our study, both oral antiviral agents were associated with similarly low rates of disease progression, hospitalization, and mortality when administered early to high-risk, non-hospitalized patients. No statistically significant differences in clinical outcomes were observed between molnupiravir and the comparator agent, nor according to vaccination status or vaccine type. As this study did not include an untreated control group, these findings should not be interpreted as evidence of a treatment effect relative to no therapy, rather, they indicate comparable clinical performance between the two agents in real-world practice. Prospective, randomized, multicenter studies remain necessary to establish the efficacy of these antivirals against an untreated or placebo comparator and to further define their comparative safety and effectiveness across different viral variants and patient subgroups.

## Figures and Tables

**Table 1:** Comparison of Demographic Characteristics, Comorbidities, and Vaccination Status Among Patients Treated with Paxlovid or Molnupiravir.

Variable	Paxlovid (N = 48)	Molnupiravir (N = 74)	*p*-Value
Age (Mean (SD))	60.21 (14.10)	63.82 (12.38)	0.138 *
Sex N (%)			
Male	20 (41.7%)	37 (50.0%)	0.367 **
Female	28 (58.3%)	37 (50.0%)	
Age categories N (%) 18–65 years >65 years	29 (60.4%) 19 (39.6%)	39 (57.2%) 35 (47.3%)	0.402 **
Smoking status No Current smoker	42 (37.2%) 6 (66.7%)	71 (62.8%) 3 (33.3%)	0.152 **
Comorbidities	39 (41.5%)	55 (58.5%)	0.374 **
Unvaccinated Vaccinated	10 (45.5%) 38 (38.0%)	12 (54.5%) 62 (62.0%)	0.517 **
Sinopharm vaccine	21 (34.4%)	40 (65.6%)	0.266 **
Pfizer–BioNTech COVID-19 vaccine	12 (42.9%)	16 (57.1%)	0.665 **
Sputnik V vaccine	6 (66.7%)	3 (33.3%)	0.081 **
AstraZeneca vaccine	0 (0.0%)	3 (100.0%)	***

*t* test *, χ^2^ test **, *p* value was not calculated because of the small number of patients in the groups ***.

**Table 2:** Comparison of Clinical Symptoms between Patients Treated with Paxlovid and Molnupiravir.

	Paxlovid (N = 48)	Molnupiravir (N = 74)	*p*-Value
Cough	25 (32.9%)	51 (67.1%)	0.061 **
Weakness and malaise	25 (29.8%)	59 (70.2%)	0.001 **
Fever	34 (35.8%)	61 (64.2%)	0.132 **
Dyspnea	3 (75.0%)	1 (25.0%)	0.138 **
Headache	4 (20.0%)	16 (80.0%)	0.078 **
Sore throat	15 (42.9%)	20 (57.1%)	0.614 **
Runny nose	10 (37.0%)	17 (63.0%)	0.751 **
Dizziness	0 (0.0%)	6 (100.0%)	***
Nausea	5 (50.0%)	5 (50.0%)	0.472 **
Vomiting	6 (85.7%)	1 (14.3%)	0.015 **
Diarrhea	9 (69.2%)	4 (30.8%)	0.033 **

χ^2^ test **, *p*-value was not calculated because of the small number of patients in the groups ***.

**Table 3:** Comparison of Comorbidity Structure between the Two Patient Groups.

	Antiviral Drug						χ^2^/Fisher’s Test *	*p*-Value
	Paxlovid		Molnupiravir		Total			
	N	%	N	%	N	%		
Comorbidities	38	79.2%	55	74.3%	93	76.2%	0.377	0.539
Cardiovascular disease	22	45.8%	47	63.5%	69	56.6%	3.704	0.054
Asthma/COPD	5	10.4%	3	4.1%	8	6.6%	*	0.261
Thyroid disease	6	12.5%	9	12.2%	15	12.3%	0.003	0.956
Diabetes mellitus	4	8.3%	17	23.0%	21	17.2%	***	0.049
Thrombophilia	4	8.3%	0	0.0%	4	8.3%	**	0.022
Chronic lymphocytic leukemia	1	2.1%	1	1.4%	2	1.6%	*	0.756
Malignancy	3	6.3%	0	0.0%	3	6.3%	**	0.059

χ^2^/Fisher’s test *, Fisher’s exact test **, Holm–Bonferroni correction ***.

**Table 4:** Laboratory Test Results and Comparison of Changes in Laboratory Parameters between Baseline (Treatment Initiation) and Follow-up in Patients Treated with Paxlovid and Molnupiravir.

Variable	Paxlovid N = 48	Molnupiravir N = 74	*p*-Value	FDR *p*
Leukocytes (4.00–10.000 10^9^/L)	−0.05 (−1.64–1.15)	−0.08 (−0.88–0.29)	0.755 ***	0.847
Neutrophils (2.00–7.50 10^9^/L)	−0.03 (−1.02–1.12)	1.05 (−6.15–3.08)	0.582 ***	0.675
Lymphocytes (0.80–4.00 10^9^/L)	0.41 (−0.01–0.94)	2.95 (0.15–8.40)	<0.001 ***	0.001
Erythrocytes (3.9–5.4 10^12^/L)	−0.01 (−0.12–0.26)	0,04 (−0.19–0.10)	0.426 ***	0.756
Hemoglobin (120–160 g/L)	0.50 (−6.00–4.25)	3.00 (−2.00–7.00)	0.124 ***	0.360
Hematocrit (0.370–0.470 L/L)	0.01 (−0.01–0.03)	0.00 (−0.01–0.01)	0.162 ***	0.675
Thrombocytes (140–400 10^9^/L)	11.00 (−7.25–45.25)	14.00 (−10.75–34.75)	0.474 ***	0.756
CRP (<5 mg/L)	−6.30 (−16.07–−2.10)	−4.80 (−13.90–−1.80)	0.529 ***	0.675
PCT (<0.05 ng/mL)	0.00 (0.00–0.00)	0.00 (0.00–0.00)	0.127 **	
Urea (2.5–8.0 mmol/L)	−0.10 (−0.80–1.32)	0.20 (−0.45–0.90)	0.434 ***	0.360
Creatinine (44–98 umol/L)	0.50 (−5.00–6.25)	1.50 (−3.00–4.00)	0.923 ***	0.675
ALT (10–40 U/L)	0.50 (−5.25–8.00)	1.00 (−1.00–2.00)	0.691 ***	0.675
AST (10–35 U/L)	−0.50 (−4.25–8.50)	2.00 (−1.75–3.00)	0.296 ***	0.099
GGT (6–42 U/L)	1.50 (−1.00–4.25)	1.00 (−2.00–2.00)	0.121 ***	0.756
Sodium (136–145 mmol/L)	2.00 (−1.00–3.25)	1.00 (−1.00–2.00)	0.093 ***	0.730
Potassium (3.5–5.5 mmol/L)	0.05 (−0.20–0.53)	0.10 (−0.08–0.20)	0.733 ***	0.370
Glucose (4.0–5.9 mmol/L)	−0.15 (−0.70–0.95)	0.05 (−0.40–0.47)	0.906 ***	0.533

IQR, Mann–Whitney U test ***, Exact *p* **. FDR—False Discovery Rate.

**Table 5:** Comparison of Chest X-ray Findings at Treatment Initiation and Follow-up.

		Antiviral Drug				χ2	*p*-Value
		Paxlovid		Molnupiravir			
		N	%	N	%		
Chest X-ray (at treatment initiation)	Pneumonia	33	40.2%	49	59.8%		
	Without pneumonia	15	37.5%	25	62.5%	0.085	0.771
Chest X-ray (follow-up)	Pneumonia	38	40.4%	56	59.6%		
	Without pneumonia	10	37.0%	17	63.0%	0.101	0.751

χ^2^ test.

**Table 6:** Hospitalization According to Vaccination Status and Type of Antiviral Drug.

		Hospitalization				
		No		Yes		
		N	%	N	%	*p*-Value
Antiviral drug	Paxlovid	46	95.8%	2	4.2%	
	Molnupiravir	70	94.6%	4	5.4%	1.000
Unvaccinated Vaccinated		22	100.0%	0	0.0%	
		94	94.0%	6	6.0%	0.590
Sinopharm vaccine		56	91.8%	5	8.2%	0.207
Pfizer–BioNTech COVID-19 vaccine		28	100.0%	0	0.0%	0.335
Sputnik V vaccine		8	88.9%	1	11.1%	0.375
AstraZeneca vaccine		3	100.0%	0	0.0%	1.000

Fisher’s exact test.

**Table 7:** Patient-Reported Symptoms Following Initiation of Antiviral Therapy with Nirmatrelvir/Ritonavir and Molnupiravir.

	Antiviral Drug						*p*-Value
	Paxlovid (N = 48)		Molnupiravir (N = 74)		Total		
	N	%	N	%	N	%	
Diarrhea	7	14.6%	4	5.4%	11	9.0%	0.109
Dysgeusia	10	20.8%	2	2.7%	12	9.8%	0.001
Headache	1	2.1%	3	4.1%	4	3.3%	0.485
Nausea	2	4.2%	2	2.7%	4	3.3%	0.646
Vomiting	3	6.3%	1	1.4%	4	3.3%	0.299

Fisher’s exact test.

## Data Availability

The datasets analysed during the current study are available from the corresponding author upon reasonable request.

## Abbreviations

The following abbreviations are used in this manuscript:

COPD	Chronic obstructive pulmonary disease
COVID-19	Coronavirus disease 2019
CYP3A4	Cytochrome P450 3A4 (hepatic enzyme involved in drug metabolism)
MERS	Middle East respiratory syndrome
NMV/r	Nirmatrelvir/ritonavir (Paxlovid)
EMA	Therapeutic management was conducted in accordance with the European Medicines Agency
PCT	Procalcitonin
ALT	Alanine aminotransferase
AST	Aspartate aminotransferase
GGT	Gamma-glutamyl transferase
RdRp	RNA-dependent RNA polymerase
RNA	Ribonucleic acid
SARS	Severe acute respiratory syndrome
SARS-CoV-2	Severe acute respiratory syndrome coronavirus 2
SD	Standard deviation
χ^2^	Chi-square test
SPSS	Statistical Package for the Social Sciences
UCCV	University Clinical Center of Vojvodina
FDR	False Discovery Rate
